# SesI May Be Associated with the Invasiveness of *Staphylococcus epidermidis*

**DOI:** 10.3389/fmicb.2017.02574

**Published:** 2018-01-04

**Authors:** Xiuqin Qi, Ye Jin, Jingjing Duan, Zhihao Hao, Shanshan Wang, Yinjuan Guo, Jingnan Lv, Longhua Hu, Liangxing Wang, Fangyou Yu

**Affiliations:** ^1^Department of Clinical Laboratory, The First Affiliated Hospital of Wenzhou Medical University, Wenzhou, China; ^2^Department of Laboratory Medicine, The Second Affiliated Hospital of Nanchang University, Nanchang, China; ^3^Department of Respiratory Medicine, The First Affiliated Hospital of Wenzhou Medical University, Wenzhou, China; ^4^Department of Clinical Laboratory, Shanghai Pulmonary Hospital, Tongji University School of Medicine, Shanghai, China

**Keywords:** *Staphylococcus epidermidis*, *sesI*, biofilm, adhesion, aggregation, virulence-associated genes

## Abstract

*Staphylococcus epidermidis* is a commensal bacterium which widely colonizes in human skin and mucous membrane and rarely causes clinically manifested infections. *S. epidermidis* surface protein I (SesI) is considered to be the major virulence factor of *S. epidermidis* infection, but its pathogenesis is not clear. Here, we demonstrated that the prevalence of *sesI* among *S. epidermidis* invasive isolates (20.8%, 26/125) was significantly higher than that among colonizing isolates (3.8%, 4/106). The positive rates of biofilm-associated genes (*aap*, *icaA*, IS*256*) and resistance-associated genes *mupA* among the *sesI*-positive isolates were significantly higher than those among *sesI*-negative isolates (*p* < 0.05). And antimicrobial susceptibility testing showed that the resistance rates of *sesI*-positive isolates to ciprofloxacin, gentamicin and trimethoprim/sulfamethoxazole were significantly higher than those among *sesI*-negative isolates. Interestingly, 80.8% (21/26) of *sesI*-positive isolates belong to ST2 determined by MLST, while ST2 was not found among any of the 99 *sesI*-negative invasive isolates, indicating that there is a strong association between carriage of *sesI* and ST2 clone. In order to further study the role of *sesI* gene in pathogenesis, the *sesI* gene mutant (*S. epidermidis* RP62AΔ*sesI*) and complementary expression strain (*S. epidermidis* RP62AΔ*sesI*-C) were successfully constructed. All experimental data indicated that *sesI* may promote *S. epidermidis* to adhere and aggregate, but it had no obvious effect on the mature stage of biofilm formation. Taken together, these results suggest that *sesI*, along with antimicrobial and other biofilm-associated genes enables *S. epidermidis* easier for colonization and adhesion and contributes to the spread of *S. epidermidis*, especially ST2 clone.

## Introduction

*Staphylococcus epidermidis*, a commensal bacterium which colonizes frequently the skin and mucous membranes of humans, is part of the human epithelial microflora and less frequently involved in clinically manifested infections (Becker et al., 2014). However, with the increasing use of indwelling or implanted medical devices including catheters, heart valves, vascular bypass grafts, nervous shunts and prosthetic implants, and the increase of multimorbid, elderly, and immunocompromised patients, *S. epidermidis* is becoming one of the major opportunistic pathogens responsible for hospital-acquired infections, especially foreign body-related bloodstream infections (Becker et al., 2014). Compared with *Staphylococcus aureus*, *S. epidermidis* possesses fewer virulence properties (Otto, 2004).

Biofilm formation is the major virulence factor of *S. epidermidis* isolates associated with device-related infections (Otto, 2004). Polysacchariden intercellular adhesin (PIA) encoded by *icaADBC* operon is the best-studied mediator for biofilm formation in *S. epidermidis* (Otto, 2004). In addition to PIA, many components have been found to be associated with biofilm formation, such as teichoic acids (TA), accumulation-associated protein (Aap), extracellular matrix-binding protein (Embp), biofilm-associated protein (Bap), phenol-soluble modulins (PSMs) (Otto, 2012). Arginine catabolic mobile element (ACME), a novel genomic island, which can enhance the capacity of this species to colonize human skin, mucosal surfaces and in-dwelling medical devices, is another important virulence-associated factor (Barbier et al., 2011; Onishi et al., 2013). The virulence-associated factors of *S. epidermidis* are not well characterized. Only a few cell-wall-associated proteins of *S. epidermidis*, including serine-aspartate repeat protein (SdrG), Embp, Ebps and AtlE, were found to have potential adhesin function binding to extracellular host matrix molecules, such as fibrinogen, fibronectin, collagen, vitronectin and elastin (Becker et al., 2014). These cell-wall-associated proteins usually display a common C-terminal cell wall sorting signal (CWS) with LPXTG [leucine–proline–variable amino acid (X)–threonine–glycine] motif. Bowden et al. reported that *S. epidermidis* surface (Ses) proteins had a similar structural organization to the previously described cell-wall-anchored proteins from *S. aureus* and other Gram-positive *cocci* and certain genes encoding Ses proteins were found more frequently in disease isolates relative to colonizing isolates (Bowden et al., 2005). Bowden et al. (2005) also found that the prevalence of *sesI* gene among invasive isolates causing bacterimia was higher than that among contaminant and colonizing isolates, but there was no significant difference between these groups. But Soderquist et al. (2009) found that *sesI* was not found among the normal *S. epidermidis* flora of healthy individuals without any healthcare association, but was found in approximately 50% of clinical isolates causing invasive infections. The association between the presence of *sesI* and invasive or colonizing isolates was not clear. The role of SesI in the pathogenicity of *S. epidermidis* is still unknown. In the present study, the aim is to investigate the prevalence of *sesI* among *S. epidermidis* clinical and colonizing isolates, microbiological and molecular characteristics of *sesI*-positive isolates and the role of *sesI* in the biofilm formation of *S. epidermidis*.

## Materials and Methods

### Bacterial Isolates

One hundred and twenty-five non-duplicate *S. epidermidis* isolates were collected from the blood and catheter of inpatients in 2002–2008 and 2012–2014 at the First Affiliated Hospital of Wenzhou Medical University, Wenzhou, China. 68 (54.4%) and 57 (45.6%) *S. epidermidis* isolates were isolated from blood and catheter respectively. The identification of *S. epidermidis* isolates was performed using Gram-staining, catalase test, coagulase test, and a VITEK-2 automated platform (bioMérieux, Marcy l’Etoile, France). *S. epidermidis* isolates from more than one set of blood culture were considered to be invasive isolates. For catheter samples, the isolates with more than 15 colonies of pure culture were regarded as invasive isolates (Liduma et al., 2012). One hundred and six *S. epidermidis* isolates obtained from the urethral orifices of healthy volunteers with no symptoms were considered to be colonizing isolates. These colonizing isolates were identified as *S. epidermidis* by 16S rRNA sequencing (Shang et al., 2005). The Ethics Committee of the First Affiliated Hospital of Wenzhou Medical University exempted this study from review because the present study focused on bacteria. Verbal informed consents were obtained from all participants.

*Escherichia coli* DH5α, *Escherichia coli* DC10B, *S. epidermidis* RP62A and pKOR1 were shown in Supplementary Files. The *S. epidermidis* RP62A is biofilm former and carries *sesI*, *icaA*, *mecA* and *aap*. And antimicrobial susceptibility testing showed that *S. epidermidis* RP62A was resistant to trimethoprim/sulfamethoxazole, clindamycin and erythromycin, while sensitive to tetracycline, penicillin, ciprofloxacin, linezolid and vancomycin. And the MLST analysis showed *S. epidermidis* RP62A belonged to ST128.

### Antimicrobial Susceptibility Testing

*In vitro* antimicrobial susceptibility testing for *S. epidermidis* invasive isolates was performed using the disk diffusion method according to the guidelines recommended by the Clinical and Laboratory Standards Institute (CLSI, 2014). Antimicrobial agents tested included ciprofloxacin (5 μg), clindamycin (2 μg), erythromycin (15 μg), gentamicin (10 μg), chloramphenicol (30 μg), linezolid (30 μg), penicillin (10 U), tetracycline (30 μg), and trimethoprim/sulfamethoxazole (1.25/23.75 μg). All antimicrobial disks were obtained from Oxoid Ltd., and *S. aureus* ATCC25923 was used as the quality control strain for antimicrobial susceptibility testing. The minimum inhibitory concentration of vancomycin was determined by the agar dilution method according to CLSI guidelines (CLSI, 2014).

### Multi-locus Sequence Typing

Multi-locus sequence typing for *S. epidermidis* invasive isolates was performed by amplifying internal fragments of seven housekeeping genes including *arcC*, *aroE*, *gtr*, *mutS*, *pyrR*, *tpiA*, and *yqiL*, as described previously (Thomas et al., 2007). Allele numbers and STs were assigned using the online database available at http://www.mlst.net.

### PCR Detection of Virulence- and Resistance-Associated Genes

All *S. epidermidis* invasive isolates were tested for the presence of *mecA* gene by PCR amplification as described previously (Murakami et al., 1991). The presence of virulence-associated genes including *aae*, *aap*, *ACME*-*arcA*, *atlE*, *bhp*, *gehD*, *icaA*, IS*256* and *sesI* was also detected by PCR amplification using previously described primers (Bowden et al., 2002; Heilmann et al., 2003; Catalanotti et al., 2005; Soderquist et al., 2009; Du et al., 2013; Cala et al., 2015). Detection of genes for mupirocin resistance (*mupA*) and chlorhexidine-based antiseptic resistance (*qacA/B*) was also performed using previously described PCR primers (Li et al., 2013).

### Construction of *S. epidermidis sesI* Mutant Strain and Its Corresponding Complemented Expression Strains

According to the results of the whole genome sequence of *S. epidermidis* RP62A, two sets of primers (Supplementary Files) containing additionally a KpnI restriction site were designed to amplify the upstream and downstream fragments of *sesI* gene, respectively. Genomic DNA (gDNA) of *S. epidermidis* RP62A was used as a template. At first, the upstream and downstream fragments of *sesI* gene were connected each other via the specific restriction sites (KpnI) by T4 DNA ligase to yield the homologous arm fragment with the deletion of *sesI* gene. Then the temperature sensitivity shuttle plasmid pKOR1 and the homologous arm fragment generated recombination reaction under the action of the BP Clonase II Enzyme, then formatted the homologous recombinant plasmid (named pK*sesI*). The recombinant plasmid (pK*sesI*) was firstly transferred into *E. coli* DH5α, then transferred into *E. coli* DC10B to modify, ultimately electroporated into *S. epidermidis* RP62A competent cells. The allelic exchange procedure was performed as described previously (Bae and Schneewind, 2006). In order to eliminate other gene mutations that may arise during the process of construction of mutant strains effecting on the results of phenotypic studies, a complementary strain was constructed. Amplifying the whole *sesI* gene and its upstream promoter and ribosome binding region (primers listed in Supplemental Files), then the gene fragments were connected to the expression vector pRB473 by the specific restriction sites, forming the recombinant complementary expression plasmid (pRB*sesI*). After modified by *E. coli* DC10B, pRB*sesI* was electroporated into the *sesI* mutant strain (RP62AΔ*sesI*). The mutant strain and complementary expression strain was verified by PCR, fluorescent quantitative PCR (RT-qPCR) and sequencing.

### RNA Extraction, cDNA Synthesis, and Quantitative Reverse Transcription-PCR (qRT-PCR)

Real time PCR was used to verify whether *sesI* gene was knocked out and complementary expression strain was constructed successfully. For RNA isolation, the overnight cultures were diluted 1:100 in TSB and grown to post-exponential phase at 37°C with shaking at 200 rpm. Total RNA was isolated and purified using a PureLink RNA Mini Kit (Invitrogen, Carlsbad, CA, United States); then was reverse transcribed into cDNA using a PrimeScript RT reagent kit (TaKaRa, Japan), according to the manufacturer’s protocol. The real-time PCR was carried out using a SsoFas EvaGreen Supermix kit (Bio-Rad, United States) with the Bio-Rad CFX96 Manager software. *S. epidermidis* RP62A wild type strain was used as a control (relative expression = 1), and *gyrB* was used as a reference gene to investigate genes of interest. RNA transcript levels were calculated by the method of delta delta Ct (ΔΔCt) (Montgomery et al., 2012). Data analysis was carried out using Bio-Rad CFX software. Each reaction was performed in triplicate. All measurements were independently conducted three times. All primers used are listed in Supplementary Files.

### Growth Curve

*Staphylococcus epidermidis* RP62A wild type strain, *S. epidermidis* RP62A, *sesI* mutant strain (*S. epidermidis* RP62AΔ*sesI*) and *S. epidermidis* RP62A *sesI* complementary expression strain (*S. epidermidis* RP62AΔ*sesI*-C) were cultured overnight in 5 ml TSB at 37°C with shaking at 200 rpm. The overnight cultures were diluted into 50 ml TSB to obtain the same starting optical density (OD) at 562 nm. The growth of each strain was monitored by Microplate Manager 6 (Bio-Rad, United States) software at 1 h intervals for a total of 12 h with sterile TSB medium as control, then tested again at 24 h. The growth curve test was repeated at least two times.

### Initial Adhesion Assay *in Vitro*

The overnight cultures were inoculated to 30 ml TSB medium according to the ratio of 1:200, and incubated at 37°C with shaking at 220 rmp until OD_562_ = 0.7∼0.8. Then the cell suspension was transferred to a microcentrifuge tube to pellet the cells, and were washed three times with sterile PBS buffer. Then PBS buffer was used to resuspend bacteria and adjust the OD_562_ = 0.6. A total of 3 ml cell suspensions was pipetted into the wells of 6-well flat bottomed polystyrene plates, which each contained a glass disk. Plates were incubated for 1h at 37°C without shaking, after which the disks were washed three times with PBS to remove the bacteria which were not adhered to the disks. Afterward, the disks were put into a sterile 50 ml microcentrifuge tube and pounded to pieces. Placed on the vortex oscillator to mix for 1 min with 15 s interval for five times, in order to remove the bacteria of adhesion on the coverlips completely. The cell suspension was diluted by 10^4^, 10^5^ and 10^6^, respectively. Hundred microliter cell suspension was cultured on blood agar plates using the spiral plating system, and detected the endpoint numbers of CFU after incubated overnight at 37°C. Assays were performed in triplicate.

### Aggregation Assay

*Staphylococcus epidermidis* PR62A wild type strain, *S. epidermidis* RP62AΔ*sesI* and *S. epidermidis* RP62AΔ*sesI*-C were incubated overnight in 5 ml sterile TSB medium at 37°C with shaking at 200 rpm, then the overnight culture medium of three strains were coated on glass slides, respectively. After drying, the gram stain were immediately performed. The aggregation ability of different strains could be observed under the microscope. Assays were performed in triplicate.

### Biofilm Formation Assay

The ability of biofilm formation of different strains was tested using a semi-quantitative adherence assay in 96-well polystyrene microtiter plates (BD Biosciences) as previously described (Shahrooei et al., 2009, 2012). Briefly, the overnight cultures were diluted 1:200 with fresh TSB medium whether or not supplemented with 4% NaCl, 1% glucose or 4% EtOH, subsequently, adjusted to obtain the same starting optical density (OD) at 562 nm. Two hundred microliter of the diluted cultures of different bacteria were pipetted into sterile 96-well polystyrene microtiter plates with three attached wells of each bacteria, then incubated overnight at 37°C without shaking. After incubation, the suspension cultures were removed from each well, a total of 200 μl sterile PBS buffer was added to the wells, and rinsed three times gently, then dried afterward. The adhered material was stained with 200 μl of a 1% (w/v) crystal violet (Sigma) solution for 10 min, and subsequently, the wells were washed three times with water and again dried. The OD_562_ of the biofilm was measured by Microplate Manager 6 (Bio-Rad, United States) software. The experiment was repeated twice.

### Scanning Electron Microscopy

Biofilm formed on glass coverslips (10 mm in diameter) was observed by scanning electron microscopy (SEM) as previously described (Pintens et al., 2008). Briefly, an overnight bacterial culture was diluted in proportion to 1:200 in TSB medium to obtain the same starting optical density (OD) at 562 nm. A glass disk was put in advance into the bottom of the sterile flat bottomed polystyrene plates (Costar 3524; Corning, NY, United States) and 1 ml of the cell suspensions was added into the wells. Then, the plates were incubated overnight at 37°C without shaking, after which each well was rinsed three times with PBS buffer, each time for 10 min with slight shaking. Biofilms formed on the glasses were fixed with 2.5% glutaraldehyde in 0.1 M sodium cacodylate buffer (pH 7.4) by incubation for 2 h at room temperature. Next the coverslips were washed three times with 0.1 M sodium phosphate buffer, each time for 15 min. Thereafter, 1% osmiumtetroxide was used for a post-fixation step for 2 h at 4°C, followed by three times washed with distilled water for 10 min each, and then dehydrated in a series of ascending ethanol baths (25, 50, 75, 95 and 100%) for 10 min each. The samples were freeze-dried for 5 h and ultimately coated with gold and palladium in an evaporator. The observations were usually performed with a scanning electron microscope (FEI Quanta 200; United States). The experiment was repeated twice.

### Triton X-100 Induced Autolysis

In order to test the potential role of *sesI* for autolysis in *S. epidermidis*, Triton X-100 induced autolysis was performed. The overnight cultures were diluted in 50 ml TSB containing 1 M NaCl, then bacterial cells were pelleted by centrifugation from early exponentially growing cultures (OD_562_ = 0.7). Afterward, the cells were washed twice with 50 ml of ice-cold water and re-suspended into 50 ml of Tris-HCl (pH 7.2) containing 0.05% (vol/vol) TritonX-100. Autolysis was monitored during incubation at 37°C by Microplate Manager 6 (Bio-Rad, United States) software at half an hour intervals for a total of 3 h. The experiment was repeated twice.

### Statistical Analysis

Chi-square or Fisher’s exact tests were used to compare the rates of biofilm-associated genes and antimicrobial resistance genes among *sesI*-positive vs. *sesI*-negative *S. epidermidis* isolates using SPSS statistical software (version 19, IBM SPSS Statistics). Statistical significance was associated with a two-sided *P*-value of <0.05. Real-time RT-PCR results were performed using GraphPad prism 6 software. A *P*-value of <0.05 was considered a significant difference.

## Results

### Prevalence of *sesI* among *S. epidermidis* Invasive and Colonizing Isolates

Twenty-six (20.8%) of 125 *S. epidermidis* invasive isolates were positive for *sesI*, while only 4 (3.8%) of 106 colonizing isolates were positive for this gene. The prevalence of *sesI* among *S. epidermidis* invasive isolates was significantly higher than that among colonizing isolates.

### Antimicrobial Susceptibility Characteristics

The resistance rates of *sesI*-positive and *sesI*-negative *S. epidermidis* invasive isolates to antimicrobial agents tested were shown in **Table 1**. All invasive isolates tested were susceptible to vancomycin. Interestingly, the resistance rates of *sesI*-positive isolates to ciprofloxacin, gentamicin and trimethoprim/sulfamethoxazole were significantly higher than those among *sesI*-negative isolates (**Table 1**).

**Table 1:** Antimicrobial resistance profiles and virulence gene carriage among *S. epidermidis* clinical isolates.

	*sesI*-positiveisolates^b^(*N* = 26)		*sesI*-negative isolates (*N* = 99)		*p*-value^c^
	No.	%	No.	%	
**Antimicrobials^a^**					
Ciprofloxacin	19	73.1	19	19.2	0.000
Chloramphenicol	5	19.2	38	38.4	0.067
Clindamycin	17	65.4	49	49.5	0.149
Erythromycin	21	80.8	81	81.8	0.902
Gentamicin	21	80.8	20	20.2	0.000
Linezolid	0	0.0	0	0.0	N/A
Penicillin	25	96.2	93	93.9	0.662
Tetracycline	5	19.2	31	31.3	0.996
SXT^b^	22	84.6	29	29.3	0.000
Vancomycin	0	0.0	0	0.0	N/A
**Virulence genes**					
*aae*	25	96.2	97	98.0	0.588
*aap*	25	96.2	56	56.6	0.000
*ACME-arcA*	9	34.6	49	49.5	0.176
*atlE*	26	100	96	97.0	0.369
*bhp*	1	3.9	23	23.2	0.026
*gehD*	13	50.0	34	34.3	0.142
*icaA*	20	76.9	28	28.3	0.000
IS*256*	18	69.2	35	35.4	0.002
**Resistance genes**					
*mupA*	8	30.8	14	14.1	0.048
*qacA/B*	13	50.0	31	31.3	0.076
*mecA*	25	96.2	89	89.9	0.316

*^a^SXT, trimethoprim/sulfamethoxazole; ^b^N, number of isolates; ^c^NA, not analyzed.*

### Prevalence of *mupA* and *qacA/B*

Although the positive rate of *qacA/B* among *sesI*-positive *S. epidermidis* invasive isolates were higher than that among *sesI*-negative isolates, there were no significant differences between the two groups. However, the prevalence of *mupA* among *sesI*-positive *S. epidermidis* invasive isolates was higher than that among *sesI*-negative isolates, which has significantly difference between the two groups (*p* < 0.05) (**Table 1**).

### Prevalence of Biofilm-Associated Genes

In the present study, the positive rate of *icaA* among *sesI*-positive *S. epidermidis* isolates (76.9%, 20/26) was significantly higher than that among *sesI*-negative isolates (28.3%, 28/99). The positive rate of *aap* among *sesI*-positive isolates was high to 96.2%, which was significantly higher than that among *sesI*-negative isolates (56.6%) (*P* < 0.05). All the *sesI*-positive *S. epidermidis* isolates were positive for *atlE*, and the prevalence of *atlE* in *sesI*-negative *S. epidermidis* isolates was 97.0%. And the prevalence of another multifunctional autolysin/adhesin gene *aae*, which encodes Aae with bacteriolytic activity that binds to fibrinogen, vitronectin and fibronectin was 96.2% vs. 98.0% in both *sesI*-positive and *sesI*-negative *S. epidermidis* invasive isolates. ACME-*arcA* was also found in both *sesI*-positive and *sesI*-negative *S. epidermidis* invasive isolates (34.6% vs. 49.5%). The prevalence of IS*256* among *sesI*-positive isolates was significantly higher than that among *sesI*-negative isolates. However, *bhp*, a gene homologous to *bap* and assumed to promote biofilm formation, was found more frequent among *sesI*-negative isolates than *sesI*-positive isolates (**Table 1**).

### Genetic Diversity of *S. epidermidis* Invasive Isolates

Distinct ST patterns were showed in **Table 2**. Among the 125 *S. epidermidis* invasive isolates, 80.8% (21/26) of *sesI*-positive isolates were belong to ST2 determined by MLST. A total of 45 STs were found among 99 *sesI*-negative isolates with ST466 being the most prevalent ST (11.1%, 11/99), followed by ST130 (8.1%, 8/99) and ST59 (7.1%, 7/99), indicating that *sesI*-negative *S. epidermidis* invasive isolates were more diverse genetically than *sesI*-positive isolates. Interestingly, ST2 was not found among the 99 *sesI*-negative invasive isolates.

**Table 2:** Sequence type (ST) distribution among *sesI*-positive and –negative *S. epidermidis* invasive isolates.

MLST	*sesI*-positive isolates (*N* = 26)		*sesI*-negative isolates (*N* = 99)		*p*-value^c^
	No.	%	No.	%	
ST2	21	80.8	0	0.0	0.000
ST5	0	0.0	1	1.0	0.607
ST14	0	0.0	1	1.0	0.607
ST16	0	0.0	2	2.0	0.465
ST218	0	0.0	2	2.0	0.465
ST47	0	0.0	2	2.0	0.465
ST20	0	0.0	5	5.1	0.242
ST21	0	0.0	1	1.0	0.607
ST57	0	0.0	2	2.0	0.465
ST59	3	11.5	7	7.1	0.455
ST89	1	3.9	1	1.1	0.305
ST125	0	0.0	5	5.1	0.242
ST130	0	0.0	8	8.1	0.134
ST206	0	0.0	1	1.0	0.607
ST210	0	0.0	5	5.1	0.242
ST226	0	0.0	2	2.0	0.465
ST227	0	0.0	2	2.0	0.465
ST234	0	0.0	2	2.0	0.465
ST235	0	0.0	2	2.0	0.465
ST263	0	0.0	3	3.0	0.369
ST267	0	0.0	1	1.0	0.607
ST419	0	0.0	1	1.0	0.607
ST466	0	0.0	11	11.1	0.075

### Initial Adhesion Assay *in Vitro*

**FIGURE 1:**
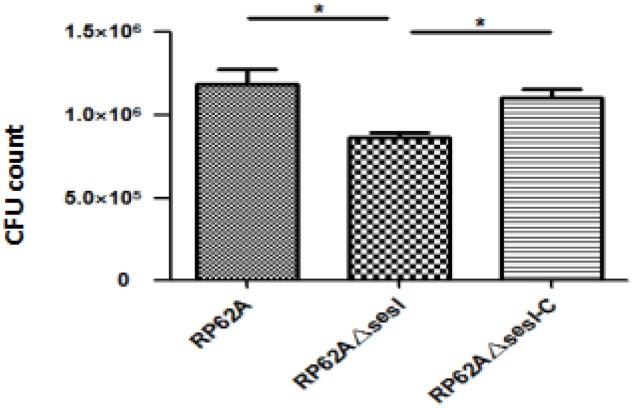
Comparison of initial adhesion ability of wild type (RP62A), isogenic *sesI* mutant (RP62AΔ*sesI*), and *sesI*-complemented (RP62AΔ*sesI*-C) strains. ^∗^Indicate *P* < 0.05.

It has been confirmed that *S. epidermidis* surface proteins have the ability to adhere human epithelial cells and extracellular matrix proteins. The i*n vitro* initial adhesion experiment was used to verify the role of *sesI* in the initial adhesion stage of bacterial biofilm formation. The ability of initial adhesion for *S. epidermidis* RP62AΔ*sesI* was significantly lower than that for *S. epidermidis* RP62A and *S. epidermidis* RP62AΔ*sesI*-C (**Figure 1**) (*p* < 0.05).

### Aggregation Assay More Dispersed

**FIGURE 2:**
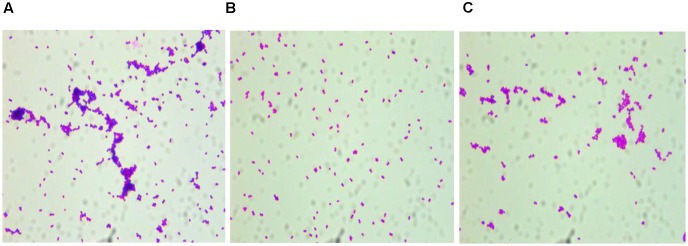
Comparison of aggregation ability of different strains. Photograph were taken by oil immersion lens. **(A)** wild type (RP62A), **(B)** the *sesI* mutant strain, **(C)** the complement strain.

The overnight cultures of all three strains tested were coated on glass coverslips after Gram staining, and observed under the microscope, showing that the distribution of *S. epidermidis* RP62A and *S. epidermidis* RP62AΔ*sesI*-C could be aggregated into blocks. However, the distribution of *S. epidermidis* RP62AΔ*sesI* was more dispersed. And the distribution density of *S. epidermidis* RP62A was significantly higher than that of *S. epidermidis* RP62AΔ*sesI* (**Figure 2**).

### Biofilm Formation Assay

**FIGURE 3:**
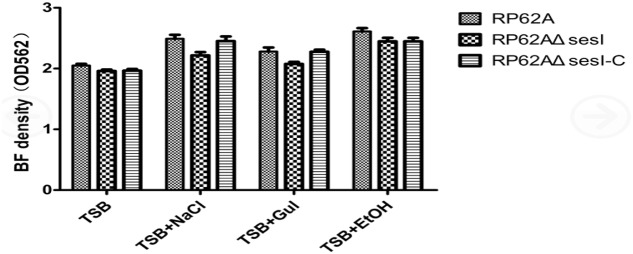
Comparison of biofilm formation in mature stage under different conditions for different strains.

The biofilm formation ability of different strains was tested using a semi-quantitative adherence assay in 96-well polystyrene microtiter plates. The results showed that there was no significant difference among *S. epidermidis* RP62A, *S. epidermidis* RP62AΔ*sesI* and *S. epidermidis* RP62AΔ*sesI*-C in the mature stage of biofilm formation whether or not under the defined predisposing conditions (added 0.5%Glu, 4%NaCl and 4% ethanol) (*P* > 0.05) (**Figure 3**).

### Scanning Electron Microscopy

Scanning electron microscopy can be used to directly and effectively observe the formation of biofilm. After a series of fixed, washed, dewatered, dried and sprayed gold for biofilm. *S. epidermidis* RP62A, *S. epidermidis* RP62AΔ*sesI* and *S. epidermidis* RP62AΔ*sesI*-C could be seen to accumulate and form microcolonies by scanning electron microscope. The microcolonies produced by all the three strains tested were overlapped with each other, forming dense clumps. It can be seen that there was no difference in the distribution and the sparsity degree of biofilm in the mature stage (**Figure 5**).

### Impact of *sesI* on *S. epidermidis* Murein Hydrolase Activity

**FIGURE 4:**
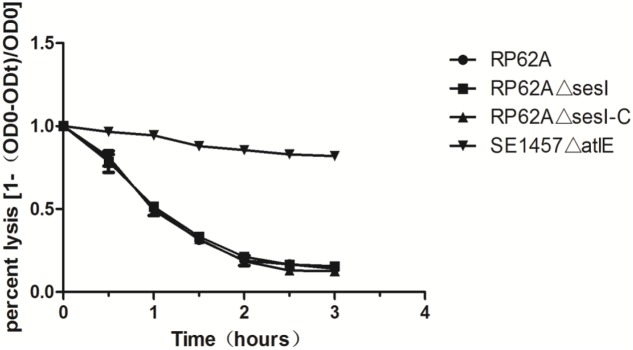
Comparison of autolytic ability of wild type (RP62A), isogenic *sesI* mutant (RP62AΔ*sesI*), and *sesI*-complemented (RP62AΔ*sesI*-C) strains.

Triton X-100 induced autolysis of bacterial cells was carried out to investigate the effect of *sesI* on autolysis in *S. epidermidis*. No difference was found between *S. epidermidis* RP62AΔ*sesI* and its parent strain in the Triton X-100 induced autolysis, while the negative control of the *atlE* knockout mutant was resistant to autolysis (**Figure 4**).

**FIGURE 5:**
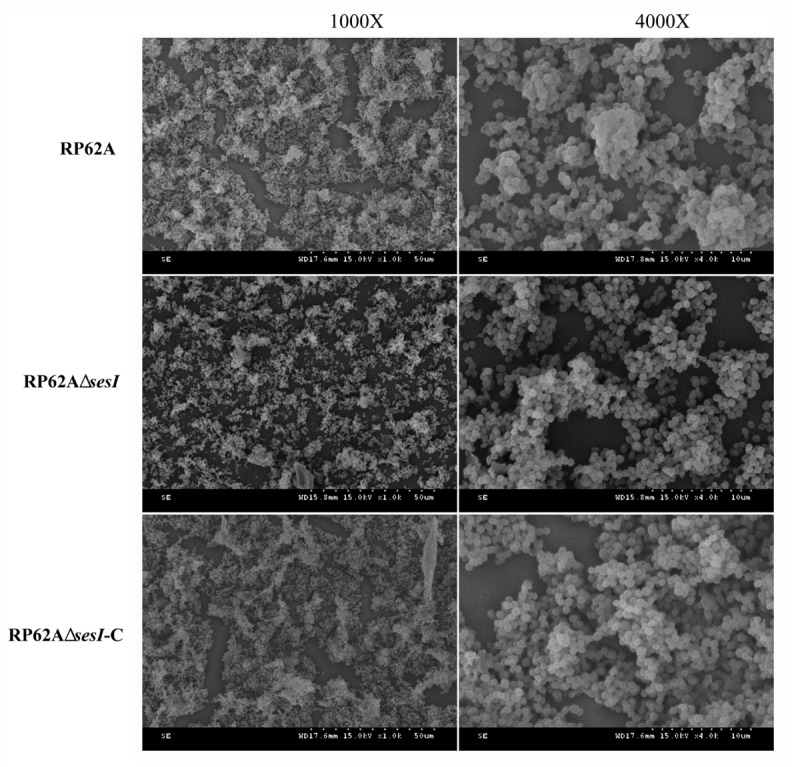
Scanning electron microscope study. Transmission electron micrograph of biofilm formation in mature stage of wild type (RP62A), isogenic *sesI* mutant (RP62AΔ*sesI*), and *sesI*-complemented (RP62AΔ*sesI*-C) strains.

## Discussion

The pathogenicity of *S. epidermidis* is mostly due to its ability to colonize implanted biomaterials and form biofilm on polymeric surfaces. The biofilm formation of *S. epidermidis* is a multifactorial process. The cell wall anchored protein (CWA) produced by Gram-positive bacteria can interact with the host extracellular matrix proteins, which can promote the adhesion function of bacteria. The cell wall associated surface protein containing the LPXTG motif can induce the surface protein to anchor to the peptidoglycan layer of the cell wall (Becker et al., 2014). Of the 11 known cell wall anchoring proteins containing the LPXTG motif, only 5 are known for their functions (Bowden et al., 2005). In the process of biofilm formation, Bhp can promote the initial adhesion and aggregation of bacteria. Aap protein is necessary for the aggregation of bacteria on the surface of polyethylene, thereby promoting the formation of a visible biofilm. SdrF and SdrG are members of the Sdr family of proteins, which are essential for the adhesion of SdrG to the human Fg matrix (Buttner et al., 2015). SesC proteins play an important role in the early stages of biofilm formation, thereby contributing to the chronic and persistent infection of the material (Khodaparast et al., 2016).

In the present study, we found that the prevalence of *sesI* among *S. epidermidis* invasive isolates was significantly higher than that among colonizing isolates. Previous study has reported that *sesI* was not found among the normal *S. epidermidis* flora of healthy individuals, but was detected in approximately 50% of invasive isolates associated with invasive infections (Soderquist et al., 2009), indicating that *sesI* may be a marker of the invasive capacity of *S. epidermidis*. For the *sesI*-positive *S. epidermidis* invasive isolates, having a higher resistance to clinically antimicrobial agents enables them to highly adapt to the nosocomial environment and to become important pathogen in healthcare setting.

Surface-active antiseptics and mupirocin are frequently used as part of comprehensive strategies designed to reduce Staphylococci colonization and infection in healthcare settings, and emergence of resistance to these antimicrobial compounds threatens hospital infection control efforts (Lepainteur et al., 2013). The *mupA* was detected by polymerase chain reaction in 63% of coagulase-negative *Staphylococci* isolates isolated from catheter-associated bloodstream infections in very preterm neonates (Lepainteur et al., 2013). In the present study, a relatively high prevalence of *mupA* found among *sesI*-positive *S. epidermidis* invasive isolates raises a challenge for the efforts to control hospital-acquired infections by decolonization of *S. epidermidis* isolates.

It has been reported that the majority of invasive *S. epidermidis* strains contain the *ica* operon in their genome. It has been verified that *icaA* is associated with biofilm formation of *Staphylococci* (Becker et al., 2014). Du et al. (2013) reported that 41.3% (43/104) of the clinical MRSE isolates isolated from blood, catheter and other sterile specimens of patients were found to carry *icaA*. In this present study, the prevalence of *icaA* among *sesI*-positive *S. epidermidis* isolates was higher relative to *sesI*-negative isolates. The accumulation-associated protein (Aap) encoded by *aap* confers intercellular adhesion and plays a role in skin colonization by its N-terminal domain A mediating adherence to human corneocytes (Macintosh et al., 2009; Salgueiro et al., 2017). The positive rate of *aap* among *sesI*-positive isolates was significantly higher than that among *sesI*-negative isolates (56.6%) (*P* < 0.05) in the present study. Previous study reported that *atlE*, encoding a surface-associated autolysin/adhesin which mediates initial adherence of bacterial cells to the polymer surfaces, was found in a total of 96.9% of invasive isolates (Heilmann et al., 1997). In the present study, more than 95% of *sesI*-positive and *sesI*-negative *S. epidermidis* isolates were positive for *atlE*. Interestingly, all *atlE*-positive isolates were also positive for another multifunctional autolysin/adhesingene *aae* encoding Aae with bacteriolytic activity that binds to fibrinogen, vitronectin, and fibronectin (Heilmann et al., 2003). Arginine catabolic mobile element (ACME), a genomic island which contributes to the enhanced capacity of *S. epidermidis* to colonize human skin and mucosal surfaces has been found in both commensal and invasive *S. epidermidis* isolates (Cherifi et al., 2013). Previous reports showed that >70% of *S. epidermidis* invasive isolates were positive for *ACME-arcA* (Cherifi et al., 2013; Du et al., 2013). In the present study, *ACME-arcA* was also found in both *sesI*-positive and *sesI*-negative *S. epidermidis* invasive isolates. The prevalence of *ACME-arcA* among *sesI*-positive isolates in the present study was similar to the result reported by Miragaia et al. (2009), in which 51% of *S. epidermidis* isolates from a widespread geographical origin were positive for *ACME*. IS*256* is involved in biofilm formation, the transcriptional regulation of resistance genes and the inactivation of global gene regulators (Maki and Murakami, 1997; Ziebuhr et al., 1999; Conlon et al., 2004). The hospital-associated *S. epidermidis* invasive isolates belonging to ST2 were all positive for *icaA* and IS*256* (Li et al., 2009). In the present study, the prevalence of IS*256* among *sesI*-positive isolates was significantly higher than that among *sesI*-negative isolates. In contrast, *bhp*, a gene homologous to *bap* and assumed to promote biofilm formation (Becker et al., 2014), was found more frequent among *sesI*-negative isolates than *sesI*-positive isolates. Taken together, our data showed that *sesI*-positive *S. epidermidis* invasive isolates harbored more virulence-associated genes relative to *sesI*-negative isolates, which is similar to the previous study (Salgueiro et al., 2017).

In the present study, majority of *sesI*-positive *S. epidermidis* invasive isolates belonged to ST2, while no *sesI*-negative invasive isolates belonged to this clone, indicating that there was strong association between carriage of *sesI* and ST2 clone. ST2 was by far the most prevalent clonal type for hospital-associated invasive *S. epidermidis* isolates (Miragaia et al., 2007; Li et al., 2009; Iorio et al., 2012; Du et al., 2013). Previous study showed the hospital-associated invasive S. *epidermidis* isolates belonging to ST2 were all positive for IS*256* and *icaA* (Li et al., 2009). Du et al. (2013) reported that IS*256* and *icaA* were detected in the majority of the predominant clinical ST2 MRSE isolates. Our study found that high prevalence of virulence-associated genes and resistance to non-lactam antimicrobials, along with a high prevalence of resistance to surface-active antiseptics used for decolonization, contribute to the spread of prevalent ST2 *sesI*-positive *S. epidermidis* clone in hospital environments.

We speculate that *icaA* and IS*256*, as well as *sesI*, are important factors which help ST2 to spread and become successfully a predominant clone in the healthcare setting.

The knockout and recovery of *sesI* gene did not affect the growth and resistance of bacteria. In order to explore the role of *sesI* in the biofilm aggregation stage, we constructed an *in vitro* aggregation experiment based on the construction of *sesI* mutant and compared the roles of the mutant and wild strains in the aggregation stage. Performing Gram staining experiment, the results showed that the distribution of mutant strains was relatively sparse, scattered and did not aggregate with each other. However, the *Staphylococcus epidermidis* RP62AΔ*sesI*-C accumulated and overlapped each other, and its ability of accumulation also returned to the wild type strain level. Therefore, it has proved *sesI* played a major role in the gathering phase. In addition, *Staphylococcus epidermidis* RP62A, RP62AΔ*sesI* and RP62AΔ*sesI*-C have been tested in semi-quantitative *in vitro* biofilms, we found that there was no significant difference in biofilm formation stage between the mutant and the wild type strain (*P* > 0.05). Biofilm formation ability of each strain was enhanced after the environmental factors changed (added 4% NaCl, 4% ethanol and 0.5% Glu), but there was no noticeable change among the three strains. By SEM, the formation of a dense and overlapping biofilm mass, indicating that during the mature stage, the formation of biofilm did not change between the mutant strain and the wild type. Our data showed that the *sesI* gene promoted the initiation adhesion and aggregation phases of biofilm formation, but not the maturation stage of biofilm formation.

Taken together, these results demonstrate that *sesI*, along with antimicrobial and other biofilm-associated genes enables *S. epidermidis* easier for colonization and adhesion and contributes to the spread of *S. epidermidis*, especially ST2 clone.

## Author Contributions

XQ, YJ, JD, ZH, SW, YG, and JL performed the laboratory measurements. FY and LW made substantial contributions to conception and design. LH, LW, and FY revised the manuscript critically for important intellectual content. LH and LW participated in experimental design and data analysis. FY drafted the manuscript. All authors read and approved the final manuscript.

## Conflict of Interest Statement

The authors declare that the research was conducted in the absence of any commercial or financial relationships that could be construed as a potential conflict of interest.

## Abbreviations

CLSI: Clinical and Laboratory Standards Institute
MLST: multi-locus sequence typing
MRSE: methicillin-resistant *Staphylococcus epidermidis*
MSSE: methicillin-susceptible *Staphylococcus epidermidis*
ST: sequence type
