# Supplementary material for: SesI May Be Associated with the Invasiveness of Staphylococcus epidermidis
Source: Front Microbiol. 2018 Jan 4;8:2574. doi: 10.3389/fmicb.2017.02574 (PMC5758504; doi:10.3389/fmicb.2017.02574)
Supplement: Supplementary file 1 [file Table_1.DOCX]

Table 1. Bacterial strains and plasmids used in this study

| Strains and  plasmids | Description | Source |
| --- | --- | --- |
| Strains |  |  |
| RP62A | Wild type, *sesI*-positive | Laboratory stock |
| RP62AΔ*sesI* | Isogenic*sesI* deletion mutant in RP62A | In this study |
| RP62AΔ*sesI*-C | *sesI* mutant complemented with pRB*sesI* | In this study |
| E. coli |  |  |
| DH5α | Clone host strain | Laboratory stock |
| DC10B | dam+1dcm^-^ΔhsdRMS endA1 recA1;clone host strain | Laboratory stock |
| Plasmids |  |  |
| pKOR1 | Shuttle cloning vector, temp sensitive (Cm^r^Amp^r^)^a^ | Laboratory stock |
| pK*sesI* | pKOR1 containing fragments 1000-bp  upstream and 1000-bp downstream of*sesI* gene, for*ses*I mutagenesis, (Cm^r^Amp^r^ ) | In this study |
| pRB473 | Shuttle cloning vector (Cm^r^) | Laboratory stock |
| pRB*sesI* | pRB473 with *sesI* and its promoter (Cm^r^) | In this study |

^a^ Cm^r^Amp^r^, chloramphenical and ampicillin resistance
