# Supplementary material for: SesI May Be Associated with the Invasiveness of Staphylococcus epidermidis
Source: Front Microbiol. 2018 Jan 4;8:2574. doi: 10.3389/fmicb.2017.02574 (PMC5758504; doi:10.3389/fmicb.2017.02574)
Supplement: Supplementary file 2 [file Table_2.DOCX]

Table 2. Primers used in this study

| Primer | Primer sequence（5'- 3'） | Note^a^ |
| --- | --- | --- |
| *sesI* us-F | GGGGACAAGTTTGTACAAAAAAGCAGGCTATCGAATGAGTCTCTAAATGCA | *attB1* |
| *sesI*us-R | GGGGTACCTTTATAAGTATCATATTCGT  AAAAT | KpnI |
| *sesI*ds-F | GGGGTACCTTTTATTTTCCTCCCAA | KpnI |
| *sesI*ds-R | GGGGACCACTTTGTACAAGAAAGCTGGGTCACATTAACATAGTACTC | *attB2* |
| *sesI-*F | TGTTCGCCTGTTTCTG |  |
| sesI-R | GCGGGTTCTGTATCAA |  |
| *sesI*-FF | TGCGATTAGGAGTTGTTT |  |
| *sesI-*RR | TATCGGTGACGTTGTGGT |  |
| *sesI*-C-F | CGGAATTCCGTCAAGATATTCATCGTTC | EcoRI |
| *sesI*-C-R | CGGGATCCCGATTTAAACCGTGATTACA | BamHI |
| *gyrB*-F | GCTGGACAGATACAAGTT |  |
| *gyrB*-R | GCTAATGCCTCGTCAATA |  |
| *sesI*-RT-F | GCTGCTAATAATACTGAAG |  |
| sesI-RT-R | CTACTACTGCTACAACTG |  |

^a^Note, Restriction endonuclease site and *attB* sequence
